# Correlation-guided Network Integration (CoNI), an R package for integrating numerical omics data that allows multiform graph representations to study molecular interaction networks

**DOI:** 10.1093/bioadv/vbac042

**Published:** 2022-06-06

**Authors:** José Manuel Monroy Kuhn, Viktorian Miok, Dominik Lutter

**Affiliations:** Computational Discovery Unit, Institute for Diabetes & Obesity, Helmholtz Zentrum München, Neuherberg, Germany; German Center for Diabetes Research (DZD), Neuherberg, Germany; Computational Discovery Unit, Institute for Diabetes & Obesity, Helmholtz Zentrum München, Neuherberg, Germany; German Center for Diabetes Research (DZD), Neuherberg, Germany; Astrocyte-Neuron Networks, Institute for Diabetes & Obesity, Helmholtz Zentrum München, Neuherberg, Germany; Computational Discovery Unit, Institute for Diabetes & Obesity, Helmholtz Zentrum München, Neuherberg, Germany; German Center for Diabetes Research (DZD), Neuherberg, Germany

## Abstract

**Summary:**

Today’s immense growth in complex biological data demands effective and flexible tools for integration, analysis and extraction of valuable insights. Here, we present CoNI, a practical R package for the unsupervised integration of numerical omics datasets. Our tool is based on partial correlations to identify putative confounding variables for a set of paired dependent variables. CoNI combines two omics datasets in an integrated, complex hypergraph-like network, represented as a weighted undirected graph, a bipartite graph, or a hypergraph structure. These network representations form a basis for multiple further analyses, such as identifying priority candidates of biological importance or comparing network structures dependent on different conditions.

**Availability and implementation:**

The R package CoNI is available on the Comprehensive R Archive Network (https://cran.r-project.org/web/packages/CoNI/) and GitLab (https://gitlab.com/computational-discovery-research/coni). It is distributed under the GNU General Public License (version 3).

**Supplementary information:**

Supplementary data are available at *Bioinformatics Advances* online.

## 1 Introduction

The increasing availability of novel omics techniques and the growing amount of produced complex biological data has generated a bottleneck at the analysis step, mainly for the lack of integration tools. The integration is challenging due to the complexity and heterogeneity of the data and the related difficulties for processing and generating summarized concrete insights (Hasin *et al.*, 2017). Biological data integration is even more difficult if one considers the vast types of available biomolecules that cover different information layers, starting from DNA modifications to RNA, phosphoproteome and metabolome. This general complexity increases by adding cellular resolution in space and time, clinical parameters and inter- and intra-organ interactions. Regarding this complexity, it is evident that new specialized tools are needed to combine and extract meaningful information from these heterogeneous datasets.

To this end, we developed Correlation-guided Network Integration (CoNI) (Klaus *et al.*, 2021), an unsupervised data-driven correlation-based tool to uncover potential interactions between features and how they are regulated between different molecular regimes. The inferred interactions and estimated regulatory variables are represented in a graph structure that allows for multiform representation. We present CoNI as an R package.

## 2 Methods

CoNI is designed to integrate two paired numerical omics datasets. A linker set $L=\left[l_{i,j}\right]$, where the row indices correspond to the linker features $i=\left(1,\ldots ,p\right)$ and the column indices $j=\left(1,\ldots ,n\right)$ correspond to the samples. Another data set, of the same samples, represents the vertex set $V=\left[v_{k,j}\right]$ where the row indices correspond to vertex features k=1,…,m*.* Features may be genes, proteins, metabolites or other continuous biological variables. Input data do not require normalization. CoNI is designed to identify and integrate features of L that are potential confounding variables for correlated feature pairs of V.

### 2.1 Integration

**Fig. 1.**
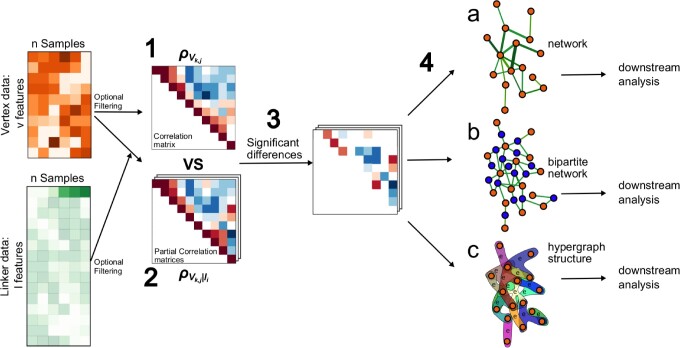
Correlation-guided Network Integration workflow. (**1**) Vertex Features are used to calculate a pairwise correlation matrix ${\rho_{v}}_{k,j}$. (**2**) Partial correlation matrices in the form $\rho_{v_{k,j}\vee l_{i}}$ are calculated using vertex pairs and linker features. (**3**) Coefficients in ${\rho_{v}}_{k,j}$ are compared to those in $\rho_{v_{k,j}\vee l_{i}}$ with Steiger tests, and confounders are selected according to significance. (**4**) Results can be represented as a network where vertex features are the nodes, and in the edges that connect them, there are one or more confounders (**a**); as a bipartite graph using vertex features as one type of node and confounders as a second type (**b**); or as a hypergraph structure (incidence matrix) where an edge can connect multiple vertices (**c**)

CoNI first calculates a correlation matrix ${\rho_{v}}_{k,j}$ for all features $v_{k}$ over the samples. A partial correlation matrix R is computed for all the triplets, consisting of two vertices and one controlling linker feature. Both matrices $\rho_{v_{k,j}\vee l_{i}}$ and ${\rho_{v}}_{k,j}$ may be sparsified by keeping only the significantly correlated pairs to retain meaningful interactions and reduce computational costs. Next, we perform significance testing comparing the difference between $\rho_{v_{k,j}\vee l_{i}}$ and ${\rho_{v}}_{k,j}$ applying Steiger test (Steiger, 1980). When we have a significant difference between $\rho_{v_{k,j}\vee l_{i}}$ and ${\rho_{v}}_{k,j}$, we consider $l_{i}$ a confounder for the vertex pairs (Klaus *et al.*, 2021). Finally, we construct a network with nodes as the correlated features of V and the edges as the identified confounding variables from L. This network structure allows multiple representations: a complex undirected weighted graph (vertices formed by V and weighted edges formed by L), a bipartite graph with L and V forming two node categories or a hypergraph where features of *L* form edges connecting multiple vertices of *V* (Fig. 1).

### 2.2 Analysis

The different output networks can subsequently be used for further analysis and visualization. CoNI uses the R package igraph for graph handling and analysis (Csardi and Nepusz, 2005). The output networks in CoNI include as default the network statistics node degree, hub score, betweenness and edge-betweenness. These or other network statistics like the node degree distribution can be used to compare networks coming from different conditions. The user can also get the shared triplets between networks of different conditions or, through a series of pre-implemented functions, count the shared linker features and summarize them based on predefined vertex classes. The results can be visualized through bar plots and stacked bar plots (see the vignette).

As CoNI allows for different network representations, each may be used for specific follow-up analysis. For example, one can apply different clustering algorithms like greedy modularity, spectral and edge-betweenness community clustering to the networks (see the vignette) to identify co-regulated communities or co-regulators. Additionally, with the hypergraph structure, one can analyze complex topological features (Klamt *et al.*, 2009).

Another pre-implemented analysis feature of CoNI is the identification of local controlling features. These features are confounders enriched for a specific subnetwork of correlated vertices (Supplementary File S1 for details). It is also possible to rank linker-features by the absolute difference between $\rho_{v_{k,j}\vee l_{i}}$ and ${\rho_{v}}_{k,j}$, where large values reflect a stronger effect on the vertex-pair relationship.

An exemplary application can be found in the package’s vignette (Supplementary File S2). This case corresponds to the analysis of the primary data of Klaus *et al.* (2021), which additionally includes another use case in the Supplementary Material. More details on how to use CoNI can be found in the help files of the R package, including a toy data example.

## 3 Results

### 3.1 Comparison on a real-world dataset

To date, a variety of data integration software exists (Subramanian *et al.*, 2020). Generally, many of these tools apply dimensionality reduction, that is, extract latent variables that capture the differences in the conditions, and features of interest are found by their contribution or correlation to these latent vectors (Argelaguet *et al.*, 2018; Picard *et al.*, 2021; Rohart *et al.*, 2017). Other tools use regularization techniques for variable selection (Wu *et al.*, 2019) or integrate multiple omics data by generating graphs or graph-like structures (Yan *et al.*, 2018). The former is typically used for disease subtyping, predicting biomarkers for distinct disease states, and clustering (Menyhárt and Győrffy, 2021). In addition, graph-based methods aim to identify and represent interactions between elements and how they are regulated between different molecular regimes (Wani and Raza, 2019).

The uniqueness of CoNI consists of its focus on identifying confounders, that is, identifying features from one dataset that potentially influence the pairwise relationships of features of another dataset and its consequent use of variable graph representations. A summary of common and different features of data integration tools is given in Supplementary Table S1. We could show that CoNI can identify relevant biological confounders (Klaus *et al.*, 2021), and we compared the results of CoNI with those of two recent unsupervised methods (Supplementary File S1).

### 3.2 CoNI on simulated datasets and comparison to other software

We use MOFA+ (Argelaguet *et al.*, 2020) to generate an artificial dataset. We then compared the results of CoNI applied on this dataset with those obtained with MOFA+, as it is also an integration tool, and iCluster+ (Shen *et al.*, 2009), another recent unsupervised tool for omics data integration. However, the unique design of CoNI to infer triplets of putative molecular interactions without prior knowledge makes it challenging to compare performance with these tools directly. Therefore, the simulated vertex data was modified using the simulated linker data to induce artificial correlations with varying degrees of change defined by the parameter *t*; the allowed absolute difference between the artificial ($\hat{p_{v}}$) and original ($p_{v}$) vertex pair correlation (Supplementary File S1). A modified vertex pair and the correlation-inducing linker feature were defined as a true positive triplet (TPT). First, we tested the ability of CoNI to recover TPTs and calculated sensitivity, specificity, and false discovery rate. We then compared the results of CoNI to those obtained by running MOFA+ and iCluster+. Like other tools, MOFA+ and iCluster+ were not designed to recover triplets; therefore, the features were searched among the top features with the highest weights from all ‘latent vectors’ (Supplementary File S1). We assumed a triplet was found if the vertex pair and linker feature were part of the features with absolute maximum weights within the same latent vector. If one or two features of the triplets were found, we considered this result a partial match.

**Fig. 2.**
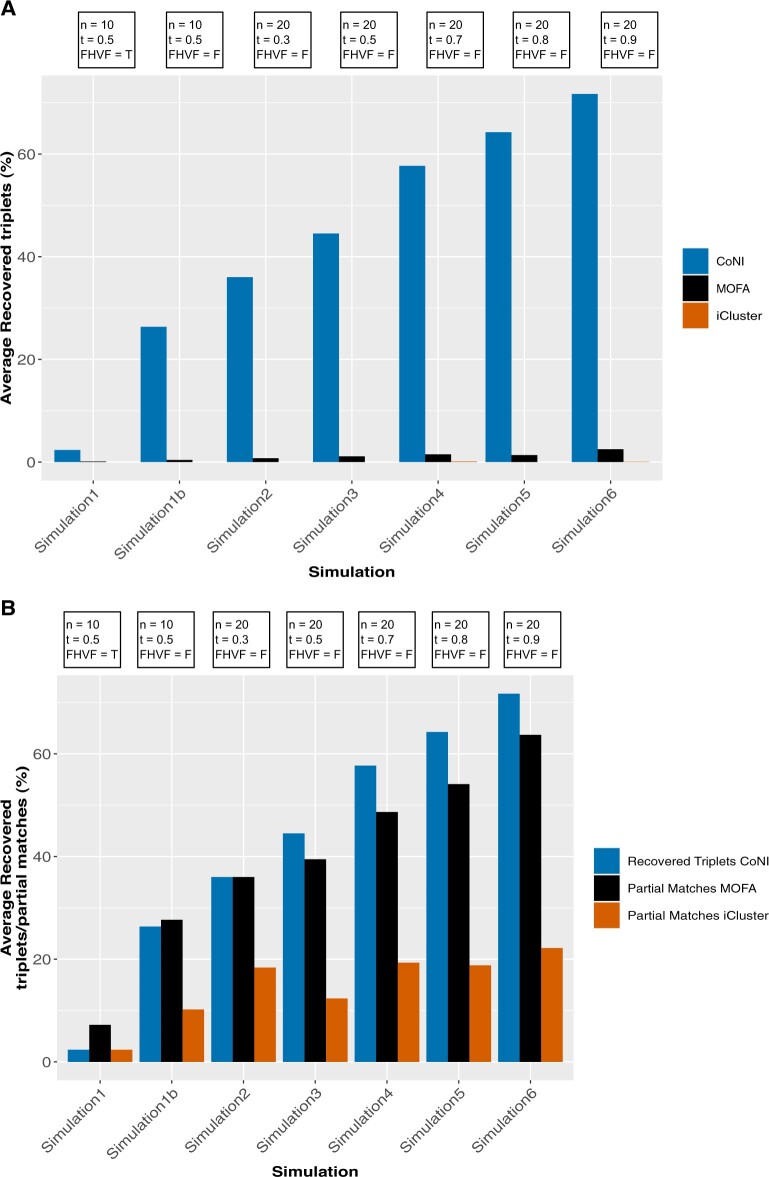
Comparison of recovered triplets and partial matches. (**A**) Barplot shows the percentage of recovered triplets for the different simulations for CoNI, MOFA+ and iCluster+. (**B**) Barplot shows the percentage of CoNI recovered triplets compared to the percentage of partial matches of MOFA+ and iCluster+. A partial match is defined as the presence of one or two features from the triplets among the features with top weights of the same latent vector. Simulation parameters can be found on top of the bars: the sample size (***n***), ‘Filter High Variance Features’ option (**FHVF**) which is either false (F) or true (T), and the absolute difference of the artificial ($\hat{p_{v}}$) and original ($p_{v}$) vertex pair correlation (***t***). Where $t\;\geq \left|\hat{p_{v}}-p_{v}\right|$

CoNI recovered between 2.4% and 71.7% of the triplets depending on the simulation parameters (Fig. 2A, Supplementary Table S10). In contrast, MOFA+ recovered <3% and iCluster+ almost no triplets in all simulations (Fig. 2A). This result is not surprising as both programs were designed for different purposes. A more even result was obtained comparing triplets to partial matches (Fig. 2B, Supplementary Table S10). The results show that CoNI can provide new insights into molecular interactions that other software do not.

**Fig. 3.**
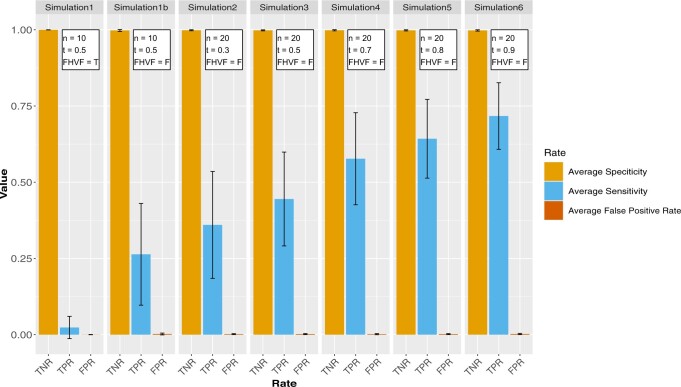
CoNI sensitivity, specificity and false discovery rate. Barplot shows average sensitivity, specificity, and false discovery rate for the CoNI results of the different simulations. Every simulation was repeated 100 times. Simulation parameters can be found on top of the bars: the sample size (*n*), ‘Filter High Variance Features’ option (FHVF), which is either false (F) or true (T), and the absolute difference of the artificial ($\hat{p_{v}}$) and original ($p_{v}$) vertex pair correlation (*t*). Where $t\;\geq \left|\hat{p_{v}}-p_{v}\right|$

Overall, CoNI showed specificity values close to one and a false-positive rate close to zero for all simulations (Fig. 3, Supplementary Table S8). Higher sensitivity values were observed for higher values of *t* (Fig. 3, Supplementary Table S8). These results indicate that CoNI is good at finding linker features with a strong effect on the relationship of the vertex pair features (Fig. 3, Supplementary Table S8). The weakest performance was observed with ten samples and the ‘Filter High Variance Features’ option (Fig. 3, Supplementary Table S8). The reason is that with this pre-filtering option, most linker features were discarded before the run. The poor performance observed with ten samples is in line with the fact that correlations perform better with more samples (Steiger, 1980). For CoNI, the same principle applies; the more samples, the better. Theoretically, there are no limitations on the number of features to run CoNI, but computational time increases as more features are given as input. The pairwise combinations of the vertex data grow in the order of *0*${(m}^{m/2})$, and the total number of combinations is then multiplied by the total number of linker features p. To run CoNI with many features is computationally intensive (Supplementary File S1). As CoNI is an open-source tool, community feedback will help to improve and extend our CoNI framework.

### 3.3 Conclusions

CoNI is an R package for unsupervised data-driven integration of two numerical omics datasets from the same samples that can be used to identify potential critical confounders of biological relevance. In addition, CoNI can represent its result in three different graph representations that allow for further analyses.

## Supplementary Material

vbac042_Supplementary_Data
