# Supplementary material for: Correlation-guided Network Integration (CoNI), an R package for integrating numerical omics data that allows multiform graph representations to study molecular interaction networks
Source: Bioinform Adv. 2022 Jun 6;2(1):vbac042. doi: 10.1093/bioadv/vbac042 (PMC9710706; doi:10.1093/bioadv/vbac042)
Supplement: vbac042_Supplementary_Data [file vbac042_supplementary_data.zip › File_S1.docx]

**Local Controlling Feature (LFCs)**

To test for an LCF, CoNI counts for every node the number of times a particular linker-feature appears in the edges located within a two-step distance. It then applies a binomial test with a probability of 1/D_net_, where D_net_ is the number of linker-features in the network.

**CoNI analysis and comparison to MOFA and sPLS**

With the data of Klaus et al. (2021), we run CoNI and two other unsupervised methods for data integration, sPLS in canonical mode from the R package MixOmics (Rohart *et al.*, 2017) and MOFA from the R package MOFA+ (Argelaguet *et al.*, 2018). To run CoNI, the filtering of the metabolites was more stringent than in Valentina et al. (2021). The metabolite data (vertex Data) was filtered based on the adjusted p-values of its pairwise correlations. As a result, a reduced list of local controlling genes (LCGs) was found compared to Klaus et al. (2021) (Table S2-3). Multiple LCGs in HFD have been previously associated with diabetes or obesity (Table S2).

We performed a KEGG and GO enrichment analysis with the genes available in the output networks of Chow and HFD using the R package ClusterProfiler (Yu *et al.*, 2012). The same was done for the genes correlated to the latent vectors separating Chow from HFD in MOFA and sPLS (Tables S4-5). Among the KEGG categories for HFD in the CoNI results, we found Glycerolipid metabolism, Glycerophospholipid metabolism, and Non-alcoholic fatty liver disease (NAFLD) (Table S4). These terms reflect the metabolic phenotype of the group of HFD mice (Klaus *et al.*, 2021). Glycerolipid metabolism was also enriched for the results of MOFA and sPLS. Other relevant terms were found only in MOFA and sPLS, including Maturity onset diabetes of the young in MOFA and Fat digestion and absorption, Insulin resistance, and Type II diabetes mellitus in sPLS. Similarly, the GO enriched terms included multiple terms associated with lipid metabolism and energy metabolism (Tables S6-7).

The results show that almost no overlap was found between the genes in the CoNI networks and those of sPLS and MOFA (Figure S1). Similarly, the enrichment results showed a slight overlap between CoNI and the other methods (Figure S2-3 and Tables S4-7). These results were expected because CoNI’s objective, rather than finding genes with an expression that separate the treatments in opposite directions, is to find per-treatment confounders from one omics dataset concerning feature pairs of a second dataset. The relevant biological functions from the genes found by CoNI indicate that CoNI can provide new disease insights not uncovered by the other methods.

**Data Simulation and CoNI performance**

To test the advantages and limitations of CoNI, we simulated data using the data-generating model of MOFA+ (Argelaguet *et al.*, 2020), using a modified version of the function ‘make_example_data’ from the R package MOFA+. The minor change of the function was done to obtain datasets with a different number of features, as the original function was designed to obtain the same number of features in all generated datasets. Every simulated dataset consisted of 200 vertex features and 10000 linker features. We used a Gaussian likelihood for both datasets and five latent vectors to model the data. Additionally, we introduced 20 artificial correlations $\hat{p_{v}},$ among the vertex pairs. To introduce the artificial correlations, we first identified 20 vertex pairs with a not-significant correlation $p_{v}$ (pval > 0.05), and for each pair, a linker feature $l$ that significantly correlated with one of the features, $p_{v_{l}}$ (pval < 0.05). Then, the expression of the linker feature was added to the expression of the vertex feature pairs. Finally, only the combinations where the modified expression led to a significant correlation $\hat{p_{v}}$ (pval < 0.05) were kept. We vary the absolute difference between the original correlation coefficient and the artificially generated coefficient for the different simulations:

$$t \geq\left| \hat{p_{v}}-p_{v} \right|$$

With *t* set to a value between 0.3 and 0.9, higher values can be interpreted as a strong effect of the linker feature on the vertex-pair relationship. The modified vertex pair and the linker feature used to modify the pair were defined as a true positive triplet (TPT). An n = 20 was used for most simulations, and only two simulations were done with an n =10, with one using the CoNI option “Filter High Variance Features” (Table S8). Every round of simulation was repeated 100 times. For every simulation, we calculated sensitivity, specificity, and false-positive rate. We assumed a conservative approach and took only the artificially generated triplets as true positives and everything else as false positives, even if it is expected to have more correlations due to the data generating model. The total number of possible triplets varied according to the number of included features in every CoNI run (Table S9).

Sensitivity increased with larger values of t (Fig. 2, Table S8). This result means CoNI is good at finding linker features with a strong effect on the relationship of the vertex pair features. Overall, CoNI showed a constant specificity, values close to one, and a false-positive rate close to zero for all simulations (Fig. 2, Table S8). The weakest performance was observed with ten samples and the “Filter High Variance Features” option (Table S8, Figure 2). The reason is that with this option, most linker features were discarded from the run. The weak performance observed with ten samples is in line with the fact that correlations perform better with more samples (Steiger, 1980). As with correlations, twenty or more samples are preferable when running CoNI.

**CoNI results on simulated data compared to other software**

CoNI was run on simulated data to test the ability to recover TPTs and compared against the results obtained by running MOFA+ and iCluster+. As MOFA+ and iCluster+ were not designed to recover triplets, the features were searched among the top ‘c’ features with the highest weights from all “latent vectors”, where ‘c’ matched the number of triplets in the output of CoNI divided by five (for every latent vector). We assumed a triplet was found if the vertex pair and linker feature were part of the features with absolute maximum weights within the same latent vector. If one or two features of the triplets were found, we considered this result a partial match.

MOFA+ was run with five factors, convergence mode slow, and the maximum number of iterations set to 10000. The default was taken for the remainder of the training options. iCluster+ was run with five factors and a tuned lambda value, obtained using the function tune.iClusterPlus (n.lambda = 89, maxiter = 50) and selecting for the model with the lowest BIC value.

**Computational requirements**

CoNI is computationally intensive with large datasets. Therefore, we recommend using a computer with at least 32 Gb of RAM, preferably a server with more resources. An example run of one treatment with 10159 genes (linker data) and 174 metabolites (vertex data) with a MacBook Pro (2019) with 32 Gb of RAM using twelve cores took 4.84 hours (split=200 see R documentation). The run time for CoNI with the simulated data on a Linux Server (92 cores, 974 GB RAM) using 80 cores took between 0.8 and 1.4 hours per run (Table S8).

**References**

Argelaguet, R. *et al.* (2018) ‘Multi-Omics Factor Analysis—a framework for unsupervised integration of multi-omics data sets’, *Molecular Systems Biology*. John Wiley & Sons, Ltd, 14(6), p. e8124. doi: https://doi.org/10.15252/msb.20178124.

Argelaguet, R. *et al.* (2020) ‘MOFA+: a statistical framework for comprehensive integration of multi-modal single-cell data’, *Genome Biology*, 21(1), p. 111. doi: 10.1186/s13059-020-02015-1.

Klaus, V. S. *et al.* (2021) ‘Correlation guided Network Integration (CoNI) reveals novel genes affecting hepatic metabolism’, *Molecular Metabolism*, p. 101295. doi: https://doi.org/10.1016/j.molmet.2021.101295.

Rohart, F. *et al.* (2017) ‘mixOmics: An R package for ‘omics feature selection and multiple data integration’, *PLOS Computational Biology*. Public Library of Science, 13(11), p. e1005752. Available at: https://doi.org/10.1371/journal.pcbi.1005752.

Steiger, J. H. (1980) ‘Tests for comparing elements of a correlation matrix.’, *Psychological Bulletin*. US: American Psychological Association, 87(2), pp. 245–251. doi: 10.1037/0033-2909.87.2.245.

Yu, G. *et al.* (2012) ‘clusterProfiler: an R Package for Comparing Biological Themes Among Gene Clusters’, *OMICS: A Journal of Integrative Biology*. Mary Ann Liebert, Inc., publishers, 16(5), pp. 284–287. doi: 10.1089/omi.2011.0118.

**Figure S1**

**Overlap genes between integration tools. (A)** HFD associated genes. **(B)** Chow associated genes.

**Figure S2**

**Overlap Biological Process GO terms between integration tools. (A)** HFD enriched terms. **(B)** Chow enriched terms.

**Figure S3**

**Overlap KEGG terms between integration tools. (A)** HFD enriched terms. **(B)** Chow enriched terms.
