# Supplementary material for: Correlation-guided Network Integration (CoNI), an R package for integrating numerical omics data that allows multiform graph representations to study molecular interaction networks
Source: Bioinform Adv. 2022 Jun 6;2(1):vbac042. doi: 10.1093/bioadv/vbac042 (PMC9710706; doi:10.1093/bioadv/vbac042)
Supplement: vbac042_Supplementary_Data [file vbac042_supplementary_data.zip › File_S2_Vignette.pdf]

CoNI

José Manuel Monroy Kuhn

2022-03-22

Contents

Introduction . . . 1
Correlation guided Network integration (CoNI) . . . 2
Run CoNI . . . 2
Create networks . . . 3
Basic network statistics . . . 4
Clustering options in igraph . . . 4
Local controlling genes (local controlling edge features) . . . 6
Genes by confounding magnitude . . . 6
Bipartite graphs . . . 8
Comparison between treatments . . . 8
Triplet comparison . . . 8
Compare metabolite-pair classes . . . 8
Compare metabolite classes per gene . . . 14

```
library(CoNI)
```

Introduction

This vignette is an introduction to `CoNI`. Following this vignette, one can analyze the data in Klaus et al. (2021). `CoNI` is run using gene expression and metabolic data for the livers of two mice cohorts, one on a high-fat diet (HFD) and the other on a chow diet (Chow).

These data is already pre-processed, that is, normalized and low-count filtered. For more information see Klaus et al. (2021)

Installation requirements `CoNI` requires a few R packages to function. Make sure they are installed before installing CoNI:

```
dependencies<-c("igraph","doParallel","cocor","tidyverse","foreach",
"ggrepel","gplots","gridExtra","plyr","ppcor","tidyr","Hmisc")

`%notin%`<-Negate(`%in%`)
for(package in dependencies){
  if(package %notin% rownames(installed.packages())){
    install.packages(package,dependencies = TRUE)
  }
}
```

```
if (!requireNamespace("BiocManager", quietly = TRUE))
    install.packages("BiocManager")
BiocManager::install("genefilter")
```

Python3 is also required to run `CoNI`. Make sure it is installed and in your path.

Correlation guided Network integration (CoNI)

Run CoNI

```
#Chow Data
data(Chow_MetaboliteData) #Metabolite data
data(Chow_GeneExpData) #Gene expression data

#HFD data
data(HFD_MetaboliteData) #Metabolite data
data(HFD_GeneExpData) #Gene expression data
```

Load data Before running `CoNI`, one has to make sure the sample names between omics datasets match (as both omic data sets should come from the same samples). If they do not match, `CoNI` will throw an error.

```
#Match rownames both omics data
rownames(Chow_MetaboliteData)<-rownames(Chow_GeneExpData)
rownames(HFD_MetaboliteData)<-rownames(HFD_GeneExpData)
#Shorten names
Chow_metabo<-Chow_MetaboliteData
Chow_gene<-Chow_GeneExpData
HFD_metabo<-HFD_MetaboliteData
HFD_gene<-HFD_GeneExpData
```

To run `CoNI`, one can decide to keep metabolites (vertex-features) based on the significance of their pairwise correlations (padjustvertexD=FALSE) or do more stringent filtering by keeping only metabolites that significantly correlate after multiple-testing adjustment (padjustvertexD=TRUE). The user can also pre-filter the genes by keeping only those that significantly correlate with at least one metabolite (correlateDFs=TRUE).

Note: This run takes around 5 hours (12 cores, 32Gb of RAM). Adjust according to the available resources.

```
#Run for Chow
CoNIResults_Chow<-CoNI(edgeD = Chow_gene,vertexD = Chow_metabo,
                       saveRaw = FALSE,filter_highVarianceEdge = TRUE,correlateDFs=TRUE,
                       padjustvertexD = FALSE, split_number = 200,
                       outputDir = "./Chow/",outputName = "CoNIChow",
                       splitedgeD = TRUE,numCores = 2,onlySgRes = TRUE)
```

To speed up this vignette, load significant Chow results:

```
#Load chow results
data(CoNIResults_Chow)
```

Note: This run takes around 5 hours (12 cores, 32Gb of RAM). Adjust according to the available resources.

```
#Run for HFD
CoNIResults_HFD<-CoNI(edgeD = HFD_gene,vertexD = HFD_metabo,
                      saveRaw = FALSE,filter_highVarianceEdge = TRUE,
                      padjustvertexD = FALSE, split_number = 200,correlateDFs=TRUE,
                      outputDir = "./HFD/",outputName = "CoNIHFD",
                      splitedgeD = TRUE,numCores = 2,onlySgRes = TRUE)
```

To speed up this vignette, load significant HFD results:

```
#Load HFD results
data(CoNIResults_HFD)
```

To speed up the computation, CoNI splits the edge Data into chunks, and it runs in parallel using the data of these chunks. Sometimes is necessary to tune 'split_number' to avoid running out of memory. For parallelization, CoNI uses the R package doParallel (Microsoft Corporation and Steve Weston, 2020). One can specify the number of cores with 'numCores'.

Internally in CoNI, the partial correlations are calculated with the R function 'pcor.test' of the package ppcor (Kim, 2015) and the Steiger tests with the function 'cocor.dep.groups.overlap' of the R package cocor (Diedenhofen and Musch, 2015).

Create networks

The user can create a network/graph and assign colors to the vertexes with the function 'generate_network'. To assign colors one has to provide a table matching vertexes and colors. The vertex names in the table have to be in the first column.

```
#Read Annotation table
data(MetaboliteAnnotation)
MetaboliteAnnotation<-assign_colorsAnnotation(MetaboliteAnnotation,col="Class")
```

```
#Generate Network
ChowNetwork<-generate_network(ResultsCoNI = CoNIResults_Chow,
                              colorVertexTable = MetaboliteAnnotation,
                              outputDir = "./",
                              outputFileName = "Chow")
#> Warning in closeness(netd_simple, mode = "all", weights = NA): At
#> centrality.c:2874 :closeness centrality is not well-defined for disconnected
#> graphs
#> Warning in centr_clo(netd_simple, mode = "all", normalized = TRUE): At
#> centrality.c:2874 :closeness centrality is not well-defined for disconnected
#> graphs
```

One can add "Class" information to the vertexes of the network, providing a data frame to the argument "Class". The first column of the data frame corresponds to the vertexes names and another column to class. This extra information is necessary for comparing treatments, where features are summarized based on the class they belong to.

```
#Generate Network Chow
ChowNetworkWithClass<-generate_network(ResultsCoNI = CoNIResults_Chow,
                              colorVertexTable = MetaboliteAnnotation,
                              outputDir = "./",
                              outputFileName = "Chow",
                              Class = MetaboliteAnnotation)
#> Warning in closeness(netd_simple, mode = "all", weights = NA): At
#> centrality.c:2874 :closeness centrality is not well-defined for disconnected
#> graphs
#> Warning in centr_clo(netd_simple, mode = "all", normalized = TRUE): At
#> centrality.c:2874 :closeness centrality is not well-defined for disconnected
#> graphs
```

```
#Generate Network HFD
HFDNetworkWithClass<-generate_network(ResultsCoNI = CoNIResults_HFD,
                              colorVertexTable = MetaboliteAnnotation,
                              outputDir = "./",
                              outputFileName = "HFD",
                              Class = MetaboliteAnnotation)
```

The networks are saved automatically (graphml format) and can be uploaded to Cytoscape for further

Table 1: Network statistics Chow

Network_statistic	Value
net_avg_pathL	4.013653
net_edge_density	0.04718945
net_transitivity	0.4093525
net_diameter	11
net_nodes_first_path_diameter	C16.1,C14.2,C14,C14.2.OH,lysoPC.a.C18.2,PC.ae.C36.3,PC.aa.C34.1,PC.aa.C36.2,PC.[illegible]
net_eigenvalue	14.38037
net_centralized_betweenessIdx	0.1102716
net_centralized_closenessIdx	0.04614072
net_centralized_degreeIdx	0.1360167

Table 2: Network statistics HFD

Network_statistic	Value
net_avg_pathL	3.377525
net_edge_density	0.05416729
net_transitivity	0.3588846
net_diameter	7
net_nodes_first_path_diameter	C10.1,Putrescine,PC.ae.C44.6,PC.aa.C40.5,PC.aa.C42.5,lysoPC.a.C20.3,C14.1,C14,C1[illegible]
net_eigenvalue	15.66564
net_centralized_betweenessIdx	0.08934598
net_centralized_closenessIdx	0.1779314
net_centralized_degreeIdx	0.1114769

exploration and visualization.

Basic network statistics

We can obtain some basic network statistics with the function NetStats.

```
library(knitr)
library(kableExtra)
kable(NetStats(Network = ChowNetworkWithClass),
      caption="Network statistics Chow") %>%
  kable_styling(full_width = FALSE)
#> Warning in centr_clo(Network, mode = "all", normalized = TRUE): At
#> centrality.c:2874 :closeness centrality is not well-defined for disconnected
#> graphs
```

```
kable(NetStats(Network = HFDNetworkWithClass),
      caption="Network statistics HFD")  %>%
  kable_styling(full_width = FALSE)
```

Clustering options in igraph

One can further make use of the networks by applying different clustering techniques available in the igraph package (Csardi & Nepusz 2006).

NOTE:For this vignette, as is small size, the networks are not displayed. Change output in the YAML metadata to html default to get a better visualization when knitting the Rmd file. Or run the code in RStudio.

```
library(igraph)
#> Warning: package 'igraph' was built under R version 4.1.2
#>
#> Attaching package: 'igraph'
#> The following objects are masked from 'package:stats':
#>
#>     decompose, spectrum
#> The following object is masked from 'package:base':
#>
#>     union
coordinates = layout_with_fr(ChowNetworkWithClass) #define layout
```

Community structure detecting based on the leading eigenvector (Spectral community)

```
Spectral = cluster_leading_eigen(ChowNetworkWithClass)
#See membership for the nodes
Spectral$membership
#>   [1]  1  1  1  4 10 10  9 10 10 10 10 10 10 10 10 10 10  5 10 10  1  9  2 12  9
#>  [26]  5  5  9  1 10  9  3 14  8  9  9  1  9  1  1  8  8  8  8  8  1 14  8  8  8
#>  [51]  8  8  5 11  9  8  5  5  5  9  9 13  5 10  5  9  9  7  9 13  2 13  6  8  8
#>  [76] 13 13  8  8  8  8  8  8  8  8  8  8  7  5  5  9  9  5  5  5  5  5  9  8  1
#> [101]  9 12  5  9  5  5 10 11 11  8  8  8  8  8  8  5  5  5 15  8  5  5  5  5  6
#> [126] 10  1  8  5  1  9  3
```

```
#Plot the network
plot(ChowNetworkWithClass,
     vertex.color=membership(Spectral),
     layout=coordinates)
```

Community structure via greedy optimization of modularity

```
greedy = cluster_fast_greedy(ChowNetworkWithClass)
#See membership for the nodes
greedy$membership
#>   [1] 6 6 6 6 5 5 2 5 5 5 5 5 5 5 5 5 5 4 5 5 6 2 8 2 2 1 1 1 6 5 2 7 6 3 6 2 6
#>  [38] 2 6 6 3 3 3 3 3 6 6 3 3 3 3 3 1 2 2 2 1 1 2 2 3 4 4 5 4 2 2 2 3 4 8 4 4 3
#>  [75] 3 4 4 3 3 3 3 3 3 3 3 3 3 1 1 1 2 2 2 1 1 1 1 2 3 1 2 2 1 2 1 1 5 2 2 3 3
#> [112] 3 3 3 3 4 4 1 4 3 1 1 1 1 4 5 6 3 4 6 6 7
```

```
#Plot the network
plot(ChowNetworkWithClass,
     vertex.color=membership(greedy),
     layout=coordinates)
```

Community structure via greedy optimization of modularity

```
betweenness = cluster_edge_betweenness(ChowNetworkWithClass,weights=NULL)
#See membership for the nodes
betweenness$membership
#>   [1]  1  1  1  1  2  2  3  2  2  2  2  2  2  2  2  2  2  4  2  2  1  5  6  7  5
#>  [26]  4  4  4  1  2  3  8  1  9  1  9  1  5  1  1  9  9  9  9  9  1  1  9  9  9
#>  [51]  9  9  4  7  7  9  4  4  9  5  9 10  4  2  4  7  9 11  9 10  6 10  4  9  9
#>  [76] 10 10  9  9  9  9  9  9  9  9  9  9  9  4  4  9  9  4  4  4  4  4  7  9  4
#> [101]  4 11  4  9  4  4  2  7  7  9  9  9  9  9  9  4  4  4  4  9  4  4  4  4  4
#> [126]  2  1  9  4  1  1  8
```

```r
#Plot the network
plot(ChowNetworkWithClass,
     vertex.color=membership(betweenness),
     layout=coordinates)
```

See igraph package for more options.

Local controlling genes (local controlling edge features)

Local controlling genes (LCGs); are genes that appear in a network region more often than expected by chance and have a potential regulatory role. To test for an LCG, CoNI counts for every node the number of times a particular gene appears in the edges located within a two-step distance. It then applies a binomial test with a probability of 1/Dnet, where Dnet is the number of genes in the network.

```r
#Chow
#Get results binomial test
LCGenes_ResultsBinomialTable_Chow<- find_localControllingFeatures(
  ResultsCoNI = CoNIResults_Chow,
  network = ChowNetworkWithClass)
#Get list local controlling genes
LCGenes_Chow<-as.character(unique(LCGenes_ResultsBinomialTable_Chow$edgeFeatures))
```

```r
#HFD
#Get results binomial test
LCGenes_ResultsBinomialTable_HFD<- find_localControllingFeatures(
  ResultsCoNI = CoNIResults_HFD,
  network = HFDNetworkWithClass)
#Get list local controlling genes
LCGenes_HFD<-as.character(unique(LCGenes_ResultsBinomialTable_HFD$edgeFeatures))
```

The user can obtain a table of the local controlling genes matching the paired metabolites and unique metabolites.

```r
#Chow
#Generate table local controlling genes
TableLCFsChow<-tableLCFs_VFs(CoNIResults = CoNIResults_Chow,LCFs = LCGenes_Chow)
#Show first two rows
TableLCFChowk<-kable(TableLCFsChow[1:2,],
                     caption="Table Local Controlling Genes") %>%
  kable_styling(full_width = FALSE)

#HFD
#Generate table local controlling genes
TableLCFsHFD<-tableLCFs_VFs(CoNIResults = CoNIResults_HFD,LCFs = LCGenes_HFD)
#Show first two rows
TableLCFHFDk<-kable(TableLCFsHFD[1:2,],
                    caption="Table Local Controlling Genes") %>%
  kable_styling(full_width = FALSE)
TableLCFHFDk
```

Genes by confounding magnitude

In addition to the LCGs, the user can obtain critical genes by ranking the network genes based on the difference of the correlation and partial correlation coefficients.

Table 3: Table Local Controlling Genes

Local Controlling edge Feature	Vertex Feature pairs
Anapc2	PC.aa.C36.2-PC.ae.C44.6,PC.aa.C38.3-PC.ae.C44.6,PC.aa.C38.5-PC.ae.C44.6,PC.aa.C4[illegible]
Appl2	PC.ae.C38.3-PC.ae.C38.4,PC.ae.C38.4-PC.ae.C40.3,PC.ae.C38.3-PC.ae.C40.4,PC.ae.C38[illegible]

```
Top10Chow<-top_n_LF_byMagnitude(CoNIResults_Chow,topn = 10)
Top10HFD<-top_n_LF_byMagnitude(CoNIResults_HFD,topn = 10)
head(Top10HFD[,c(1:3,ncol(Top10HFD))])
#>   Feature_1_vertexD Feature_2_vertexD Feature_edgeD difference
#> 1        PC.ae.C42.0        PC.ae.C42.2         Lpin2   1.737202
#> 2        PC.ae.C42.5      SM..OH..C24.1        Ppp6r1   1.644208
#> 3        PC.aa.C38.1                 H1       Tmem205   1.638176
#> 4        PC.ae.C42.0        PC.ae.C42.2          Rac1   1.632434
#> 5                 C3                Phe       Rapgef4   1.620366
#> 6        PC.aa.C42.0        PC.aa.C42.4         Tmed1   1.609743
```

In the results of HFD, we can see that the largest difference between coefficients is for the triplet "PC.ae.C42.2", "PC.ae.C42.0" and "Lpin2". One can visualize the effect of "Lpin2" on the metabolites by fitting two linear models on standardize data and plotting the results. One model we use the metabolite expression and in the other the residuals from the linear models between the gene and each of the metabolites. In one linear model, the slope is the correlation coefficient between metabolites, and in the other, the partial correlation coefficient.

```
plotPcorvsCor(ResultsCoNI = CoNIResults_HFD,edgeFeature = "Lpin2",
              vertexFeatures = c("PC.ae.C42.2","PC.ae.C42.0"),
              vertexD = HFD_metabo,edgeD = HFD_gene,
              label_edgeFeature = "Gene",plot_to_screen = TRUE,
              outputDir = "./")
```

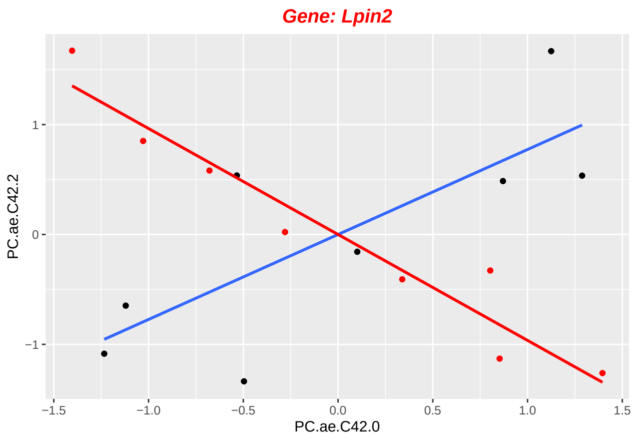

The user can also explore all the metabolite pairs that form triplets with the gene in question.

```
plotPcorvsCor(ResultsCoNI = CoNIResults_HFD,edgeFeature = "Lpin2",
              vertexD = HFD_metabo,edgeD = HFD_gene,
              label_edgeFeature = "Gene",plot_to_screen = TRUE,
              outputDir = "./")
```

Bipartite graphs

An alternative network representation is a bipartite graph. In this type of graph, there are two types of nodes. For the example in this vignette, one node type are genes (linker-features, inside the edges in the previous network) and the other metabolites (vertex_features, nodes in the previous network)

```
#Chow
ChowBipartiteGraph<-createBipartiteGraph("./TableForNetwork_Chow.csv",MetaboliteAnnotation)
#> Warning in closeness(netd, mode = "all", weights = NA): At
#> centrality.c:2874 :closeness centrality is not well-defined for disconnected
#> graphs
#> Warning in centr_clo(netd, mode = "all", normalized = TRUE): At
#> centrality.c:2874 :closeness centrality is not well-defined for disconnected
#> graphs
#Save bipartite graph
write.graph(ChowBipartiteGraph,file="./Chow_bipartite.graphml",format="graphml")

#HFD
HFDBipartiteGraph<-createBipartiteGraph("./TableForNetwork_HFD.csv",MetaboliteAnnotation)
#Save bipartite graph
write.graph(HFDBipartiteGraph,file="./HFD_bipartite.graphml",format="graphml")
```

The resulting bipartite graph can be uploaded in Cytoscape for further visualization and exploration.

It is also possible to obtain a hypergraph incidence matrix instead of a bipartite graph

```
Chow_HypergraphIncidenceM<-createBipartiteGraph("./TableForNetwork_Chow.csv",
                                                MetaboliteAnnotation,
                                                incidenceMatrix = TRUE)
#> Warning in closeness(netd, mode = "all", weights = NA): At
#> centrality.c:2874 :closeness centrality is not well-defined for disconnected
#> graphs
#> Warning in centr_clo(netd, mode = "all", normalized = TRUE): At
#> centrality.c:2874 :closeness centrality is not well-defined for disconnected
#> graphs
```

Comparison between treatments

Triplet comparison

Exact triplet matching (metabolite pair - gene) can be done as follows:

```
Compare_Triplets(Treat1_path = "./TableForNetwork_Chow.csv",
                 Treat2_path = "./TableForNetwork_HFD.csv",
                 OutputName = "Shared_triplets_HFDvsChow.csv")
#> [1] Vertex_1 Vertex_2 Edge
#> <0 rows> (or 0-length row.names)
```

No triplets were shared between treatments, reflecting the strong metabolic differences between HFD and Chow.

Compare metabolite-pair classes

Some genes are shared between the resulting CoNI networks of Chow and HFD. These genes do not share the same metabolite pairs but they might share similar metabolite classes.

```
(LCP_sharedGene_HFDvsChow<-Compare_VertexClasses_sharedEdgeFeatures(
                          Treat1_path = "./TableForNetwork_HFD.csv",
```

```
                           Treat2_path = "./TableForNetwork_Chow.csv",
                           OutputName = "Comparison_LipidClassesPerGene_HFDvsChow.csv",
                           Treat1Name = "HFD",
                           Treat2Name = "Chow"))
#>    EdgeFeature VertexClassPair HFD Chow
#> 1       Gm4553           BA_PC   2    0
#> 2       Gm4553           AA_BA   5    0
#> 3       Gm4553           AA_PC   2    0
#> 4       Gm4553          BA_LPC   0    1
#> 5       Hnrnpm           BA_PC   3    0
#> 6       Hnrnpm           BA_BA   1    0
#> 7       Hnrnpm         ACC_LPC   0    1
#> 8         Xpo7          LPC_PC   1    0
#> 9         Xpo7           PC_PC   4    0
#> 10        Xpo7          ACC_PC   0    1
#> 11        Tap1           PC_PC   1    0
#> 12        Tap1          ACC_SM   0    1
#> 13        Eya3           PC_PC   1    1
```

The results show that the shared genes also show a shift in the metabolite classes they putatively interact with.

One can create a barplot of one of the shared genes to compare the metabolite-pair profile between treatments.

```
create_edgeFBarplot(CompTreatTable = LCP_sharedGene_HFDvsChow,
                    edgeF = "Gm4553",
                    treat1 = "HFD",
                    treat2 = "Chow",
                    factorOrder = c("HFD","Chow"),
                    col1="#E76BF3",
                    col2 = "#F8766D",EdgeFeatureType = "Gene")
```

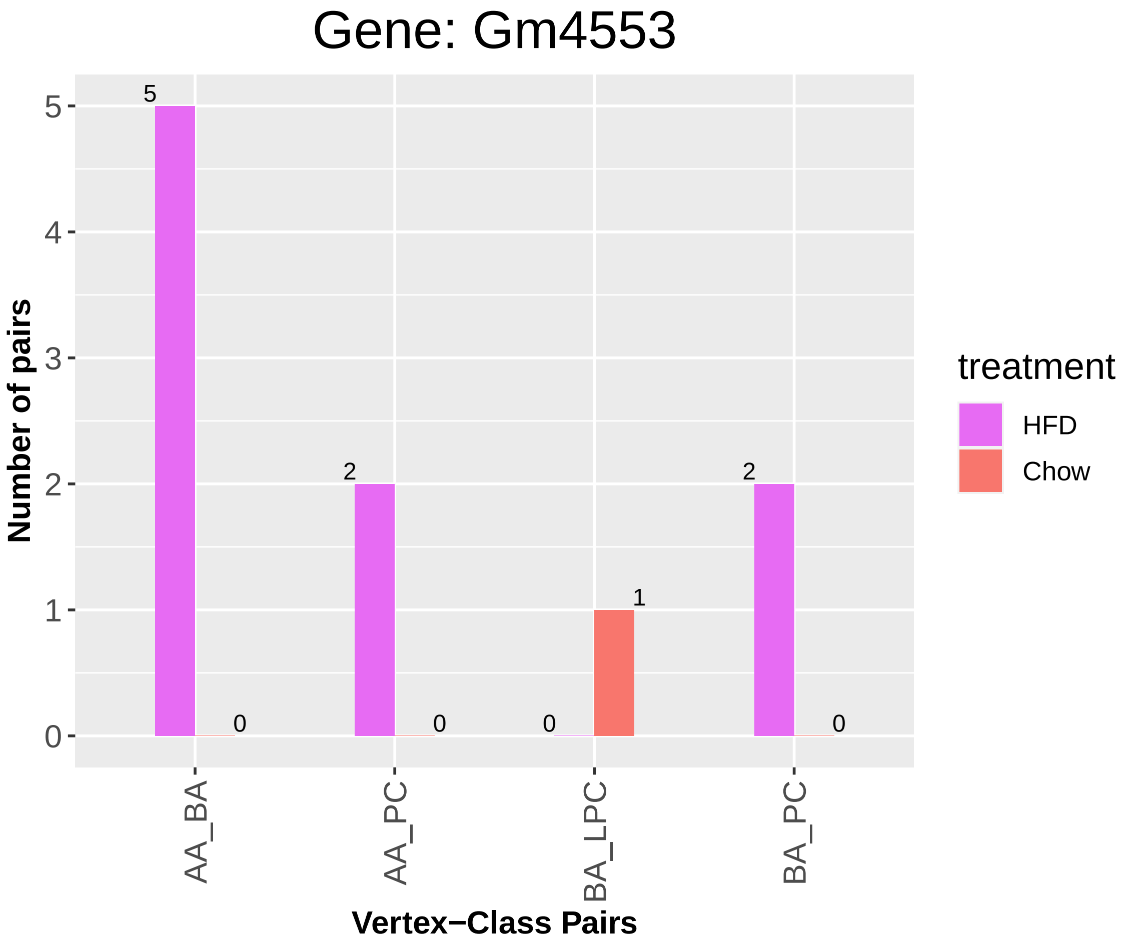

If the user wants to explore the global metabolite-pair profile of the shared genes, it can use the function create_GlobalBarplot as follows.

```
(HFDvsChow_GlobalLipidProfile<-create_GlobalBarplot(CompTreatTable = LCP_sharedGene_HFDvsChow,
                                                     treat1 = "HFD",
                                                     treat2 = "Chow",
                                                     factorOrder = c("HFD","Chow"),
                                                     col1="#E76BF3",
                                                     col2 = "#F8766D",
                                                     maxpairs = 1,
                                                     szggrepel = 6,
                                                     szaxisTxt = 15,
                                                     szaxisTitle = 15,
                                                     xlb = "Metabolite-pair classes"))
```

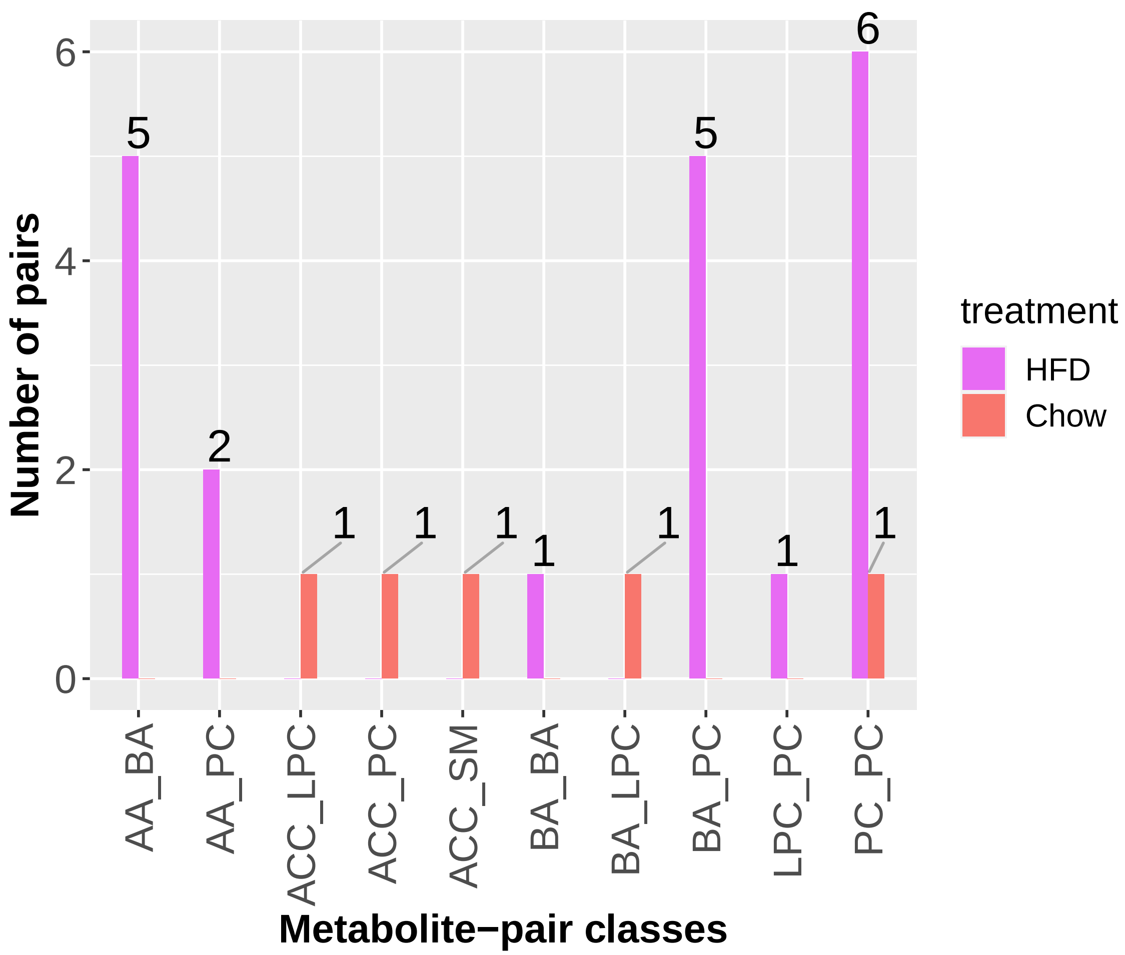

The user can also explore the global metabolite-pair profile of the shared genes per treatment but showing how every gene contributes to the profile.

```
create_stackedGlobalBarplot_perTreatment(CompTreatTable = LCP_sharedGene_HFDvsChow,
                                         treat = "HFD",
                                         max_pairsLegend = 1,
                                         xlb = "Metabolite-class-pairs",
                                         szTitle = 20,
                                         szggrepel = 6,
                                         szaxisTxt = 15,
                                         szaxisTitle = 15)
#> 6

create_stackedGlobalBarplot_perTreatment(CompTreatTable = LCP_sharedGene_HFDvsChow,
                                         treat = "Chow",
                                         max_pairsLegend = 1,
                                         ylim = 3,
                                         xlb = "Metabolite-pair classes",
                                         szTitle = 20,
                                         szggrepel = 4,
                                         szaxisTxt = 15,
                                         szaxisTitle = 15)
#> 1
```

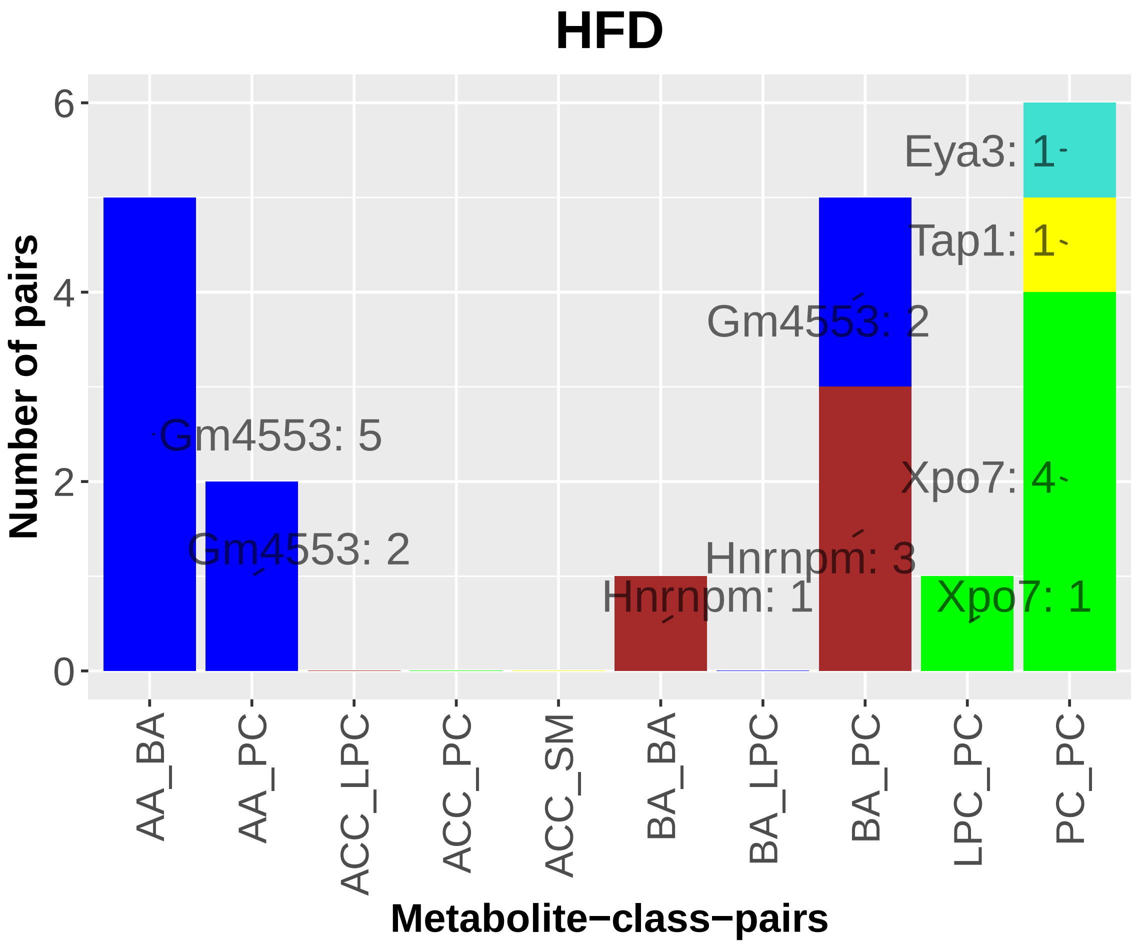
HFD
Number of pairs
Metabolite−class−pairs
0
2
4
6
AA_BA
AA_PC
ACC_LPC
ACC_PC
ACC_SM
BA_BA
BA_LPC
BA_PC
LPC_PC
PC_PC
Gm4553: 5
Gm4553: 2
Hnrnpm: 1
Gm4553: 2
Hnrnpm: 3
Xpo7: 1
Eya3: 1
Tap1: 1
Xpo7: 4

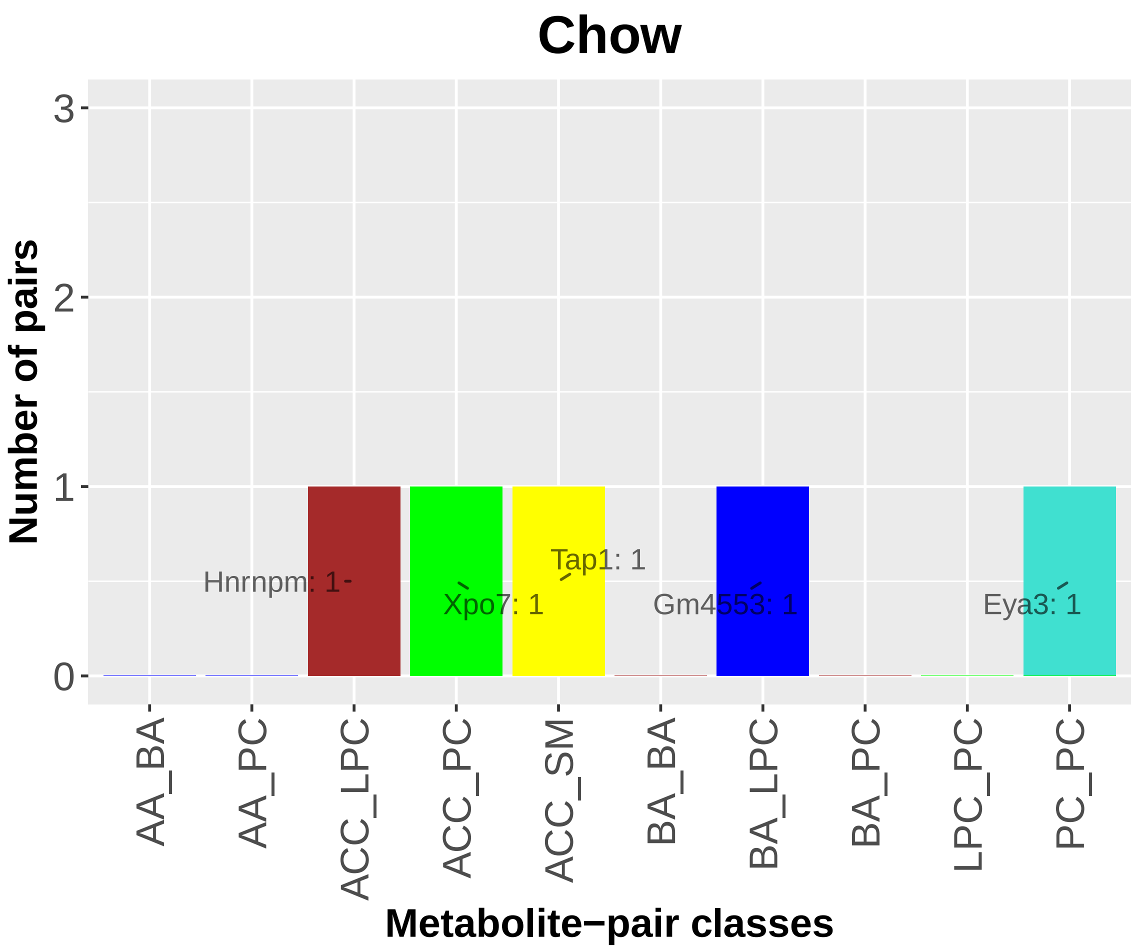

It is possible to obtain the previous plots as a single figure and with the same y-axis range.

```
HFDvsChow_StackedLipidProfile<-getstackedGlobalBarplot_and_Grid(
                                 CompTreatTable = LCP_sharedGene_HFDvsChow,
                                 xlb = "Metabolite-pair classes",
                                 Treat1 = "HFD",
                                 Treat2 = "Chow",
                                 szTitle = 20,
                                 szggrepel = 6,
                                 szaxisTxt = 15, szaxisTitle = 15)
plot(HFDvsChow_StackedLipidProfile)
```

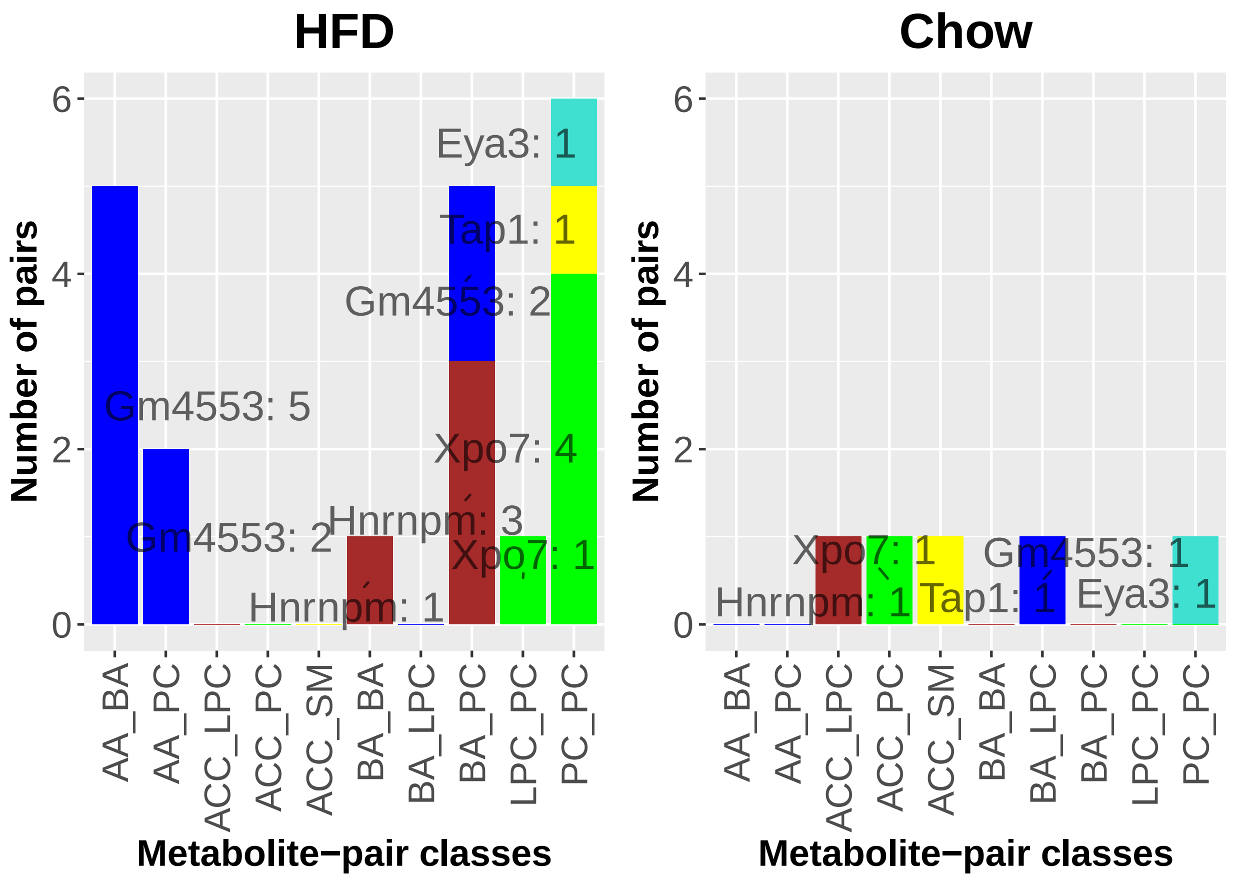

Compare metabolite classes per gene

The user can explore the results from the perspective of the genes and counting the metabolites per class.

```
HFD_vs_Chow_LCP_Gene<-getVertexsPerEdgeFeature_and_Grid(LCP_sharedGene_HFDvsChow,"HFD","Chow",
                                                        Annotation=MetaboliteAnnotation,
                                                        ggrep=FALSE,
                                                        small = TRUE,
                                                        szTitle = 20,
                                                        szaxisTxt = 15,
                                                        szaxisTitle = 15)
plot(HFD_vs_Chow_LCP_Gene)
```

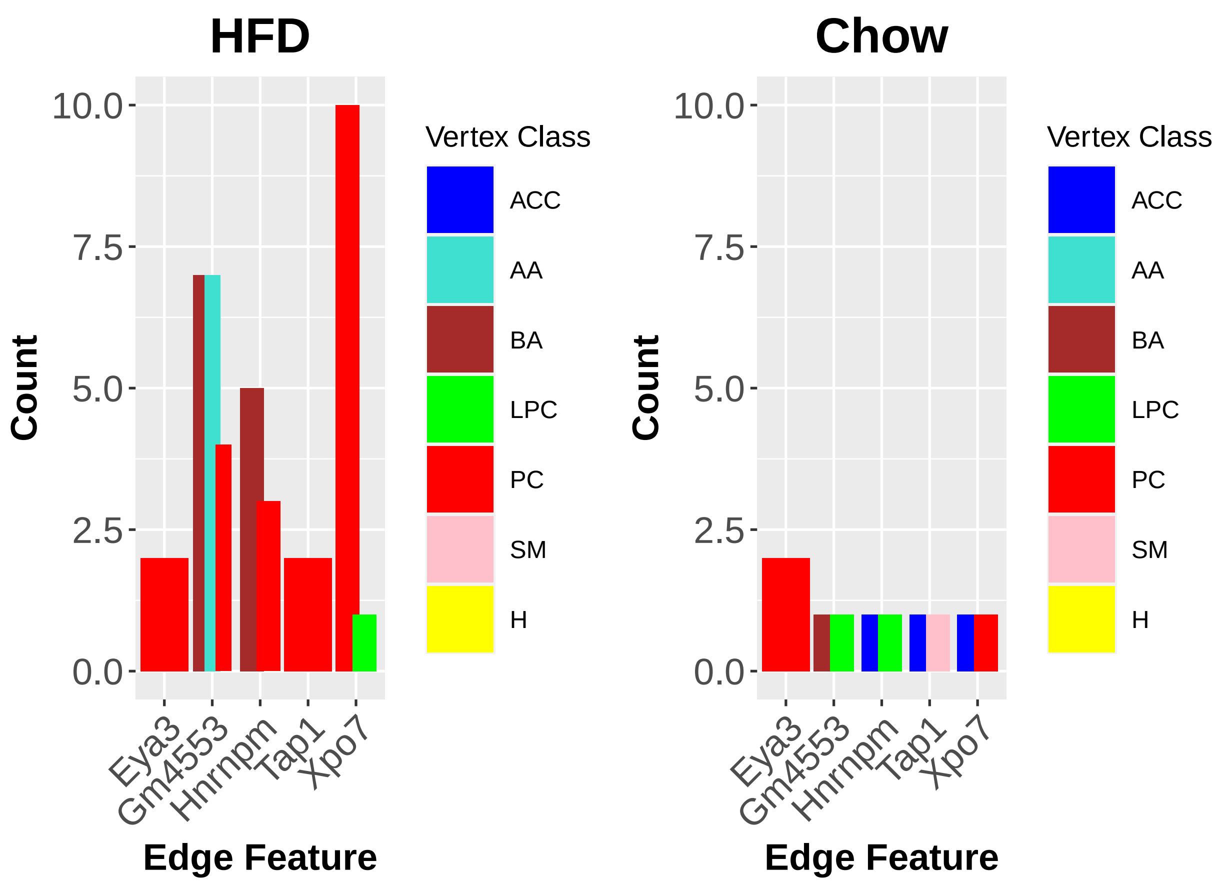

HFD
Chow
Count
10.0
7.5
5.0
2.5
0.0
Vertex Class
ACC
AA
BA
LPC
PC
SM
H
Eya3
Gm4553
Hnrnpm
Tap1
Xpo7
Edge Feature
